# Cytokine Signature in End-Stage Renal Disease Patients on Hemodialysis

**DOI:** 10.1155/2017/9678391

**Published:** 2017-07-27

**Authors:** Danyelle Romana Alves Rios, Melina Barros Pinheiro, Wander Valadares de Oliveira Junior, Karina Braga Gomes, Andréa Teixeira Carvalho, Olindo Assis Martins-Filho, Ana Cristina Simões e Silva, Luci Maria Sant'Ana Dusse

**Affiliations:** ^1^Campus Centro Oeste, Federal University of Sao Joao del-Rei, Divinópolis, MG, Brazil; ^2^Department of Clinical and Toxicological Analysis, Faculty of Pharmacy, Federal University of Minas Gerais, Belo Horizonte, MG, Brazil; ^3^Laboratório de Biomarcadores de Diagnóstico e Monitoração, Centro de Pesquisas René Rachou, Fundação Oswaldo Cruz, Belo Horizonte, MG, Brazil; ^4^Department of Pediatrics, Interdisciplinary Laboratory of Medical Investigation, Faculty of Medicine, Federal University of Minas Gerais, Belo Horizonte, MG, Brazil

## Abstract

Hemodialysis is a modality of blood filtration in which accumulated toxins and water are removed from the body. This treatment is indicated for patients at the end stage of renal disease. Vascular access complications are responsible for 20–25% of all hospitalizations in dialyzed patients. The occurrence of thrombosis in the vascular access is a serious problem that may severely compromise or even make the hemodialysis impossible, which is vital for the patient. The aim of this study was to investigate inflammatory profile in patients undergoing hemodialysis as well as the association between these alterations and vascular access thrombosis. A total of 195 patients undergoing hemodialysis have been evaluated; of which, 149 patients had not experienced vascular access thrombosis (group I) and 46 patients had previously presented this complication (group II). Plasma levels of cytokines including interleukin (IL-) 2, IL-4, IL-5, IL-10, TNF-*α*, and IFN-*γ* were measured by cytometric bead array. Our results showed that patients with previous thrombotic events (group II) had higher levels of the IL-2, IL-4, IL-5, and IFN-*γ* when compared to those in group I. Furthermore, a different cytokine signature was detected in dialyzed patients according to previous occurrences or not of thrombotic events, suggesting that elevated levels of T-helper 1 and T-helper 2 cytokines might, at least in part, contribute to this complication.

## 1. Introduction

Hemodialysis (HD) requires a well-functioning vascular access that allows sufficient blood flow to achieve adequate clearance and blood dialysis. Vascular access complications increase morbidity and are responsible for 20–25% of all hospitalizations in dialyzed patients; of which, approximately 85% of the cases are due to thrombosis [1, 2]. Although substantial progress has been achieved in preventing thrombotic complications in various groups of patients, the mechanisms of thrombosis during hemodialysis still require clarification.

End-stage renal disease (ESRD) and HD itself lead to an inflammatory status induced by multiple factors, including uremic syndrome per se, heart failure, vascular access infections, bioincompatible dialysis solutions, the accumulation of advanced glycation products, progressive decrease of glomerular filtration rate [3–5], and blood-membrane interaction [6]. Chronic inflammation as a major determinant of “dialysis syndrome” is considered as the main factor of morbidity and mortality in dialysis patients. Therefore, the immune system is recognized as one of the most important therapeutic targets to reduce morbidity and mortality in uremic and dialysis patients [7].

Inflammation is characterized by the enhanced production of C-reactive protein (CRP) and other inflammatory mediators, including T-helper 1 (Th-1) cytokines such as tumor necrosis factor-*α* (TNF-*α*), interleukin (IL-) 6, and IL-2 as well chemokines such as IL-8 [8]. Although viewed separately, coagulation and inflammation are highly related. Both biological processes interact and cooperate for the organism response to injury and invasion by pathogens. It is known that the coagulation proteins, besides acting in cascade promoting the formation of the fibrin clot, also have an isolated action in other pathophysiological processes. In this way, the tissue factor (TF), factor VII, factor X, and thrombin have an important role in inflammatory process [9]. It is also known that CRP, TNF-*α*, and IL-1 stimulate TF expression by monocytes and endothelial cells [10, 11]. Dysregulation of any component of these systems can affect the entire balance, resulting in a wide range of illnesses that may result in variable degrees of inflammation and thrombosis [12].

Inflammation in dialysis patients may vary over time and during this process. Anti-inflammatory and modulatory cytokines can also be produced as an attempt to control this process, such as IL-4, IL-5, and IL-10. IL-10 is secreted by lymphocytes of the T-helper 2 (Th2) subtype and also in large amounts by macrophages [13]. IL-10 is an anti-inflammatory cytokine with potent deactivating properties on both macrophages and T cells. IL-10 inhibits several cellular processes responsible for plaque progression, rupture, or thrombosis, including nuclear factor-*κ*B (NF-*κ*B) activation, metalloproteinase production, TF and cyclooxygenase-2 expression, and cell death [13, 14]. It is known that IL-4 can modulate the already existing inflammatory process and can also induce the expression of t-PA that contributes to the activation of plasminogen in the patients who presented thrombotic events [15].

The aim of this study was to evaluate plasma levels of Th-1 and Th-2 cytokines in patients undergoing hemodialysis and the association of the cytokine profile with thrombotic complications in vascular access.

## 2. Methods

### 2.1. Study Design

This was an observational cross-sectional study.

### 2.2. Patients

The present study included HD patients for more than six months (*n* = 195) with arteriovenous fistula, aged between 18 and 70 years, selected from two dialysis centers in Belo Horizonte, MG, Brazil, according to the exclusion and inclusion criteria.

The HD patients were allocated into two subgroups, according to the occurrence or not of a previous episode of vascular access thrombosis (VAT) (with and without VAT). HD patients with VAT (group II) consisted of 46 individuals whose functioning dialysis access had, at least, one previous episode of clinically evident thrombotic occlusion. Thrombotic occlusion was defined by the absence of blood flow and the impossibility to use the access for dialysis. The remained HD patients consisted of 149 subjects who have not experienced this complication (group I).

All patients required regular HD sessions for 3 to 4 hours, three times a week. Blood flow was usually 300–450 mL/min with a dialysate flow at a constant rate of 500 mL/min. Patients were dialyzed either with low-flux polysulphone membranes or high-flow polysulphone membranes with bicarbonate-buffered dialysate. All patients received regular doses of standard heparin (100 to 150 UI/kg) before a hemodialysis session.

Exclusion criteria were hemodialysis patients receiving oral anticoagulation therapy or oral contraceptives, with prior history of arterial or venous thrombosis (except those with VAT), with acute or chronic hepatic disease, autoimmune and malignant diseases, vasculitis, acute infections, clinical instabilities, a history of renal transplantation, and HIV-positive and pregnant women.

### 2.3. Ethical Considerations

The ethics committee of our institution approved the study, and informed consent was obtained from all participants. The research protocol did not interfere with any medical recommendations or prescriptions.

### 2.4. Variables

A detailed history, clinical variables [age, gender, body mass index (BMI), predialysis blood pressure levels, etiology of ESRD, presence of diabetes or not, type of vascular access, time on hemodialysis, and interdialytic weight gain], and laboratorial parameters including known factors affecting thrombosis such as platelet numbers, hsCRP, leukocytes, and erythrocytes, as well as nutritional parameters such as albumin and normalized protein catabolic rate (nPCR) of each included patient were recorded retrospectively in a computer-specific data bank.

After informed consent, all subjects were submitted to blood collection for the determination of plasma levels of cytokines.

### 2.5. Cytometric Bead Array for Cytokine Measurements

Blood samples were drawn in EDTA-K_3_ 1.8 mg/mL (Vacuette®), using a 16G needle, from HD vascular access prior to dialysis procedure at the first dialysis session of the week and before heparin administration. Samples were centrifuged at 2500*g* for 20 min at 4°C to obtain plasma, which was stored at −70°C until analysis.

Cytokine plasma levels acquisition and analysis were performed in a dual-laser FACSCalibur™ flow cytometer (BD Biosciences Pharmingen, San Jose, CA, USA) using the BD Bioscience CBA software. IL-2, IL-4, IL-5, IL-10, IFN-*γ*, and TNF-*α* were determined using the Human Th1/Th2 cytometric bead array method (BD Biosciences Pharmingen, USA), as previously described by Pinheiro et al. [16].

### 2.6. Analysis of Plasma Levels of Cytokines and Cytokine Signature

Plasma levels of cytokines were analyzed as the mean fluorescence intensity (MFI) provided by the CBA immunoassay. Additionally, the analysis referred as “cytokine signature” was also performed as previously proposed by Luiza-Silva et al. and Pinheiro et al. [16, 17]. Briefly, the global median value for each cytokine was calculated taking the whole data universe from all patients. The global median cut-off was used as the cut-off edge to tag each patient as they display “low levels” (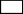 for all cytokines) and “high levels” (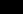 for all cytokines). The frequency (%) of patients showing “high cytokine levels” was calculated, and the “cytokine signature” assembled as the ascendant frequencies in order to identify changes in the overall cytokine patterns between the two studied groups.

### 2.7. Statistical Analysis

Variables were checked for Gaussian distribution by the Shapiro-Wilk test. Non-Gaussian data were expressed as median and interquartile range (25th percentile and 75th percentile). Demographic, clinical characteristic and plasma levels of cytokines, expressed as MFI, were compared among groups by Mann–Whitney *U* test. All analyses were performed using the program GraphPad PRISM (version 5.0), and significant differences, in all cases, are considered at *p* < 0.05. Additional strategy of data analysis was used to compare the ascendant profile referred as “cytokine signature.” Relevant differences were considered when the frequency of patients with “high cytokine levels” was above the 50th percentile.

## 3. Results

Clinical variables, laboratorial and nutritional parameters, did not differ between group I and group II, as displayed in Tables 1 and 2. Age, gender, body mass index (BMI), predialysis blood pressure levels, etiology of ESRD, presence of diabetes or not, type of vascular access, time on hemodialysis, and interdialytic weight gain were similar in both groups (*p* > 0.05 for all comparisons).

**Figure 1:**
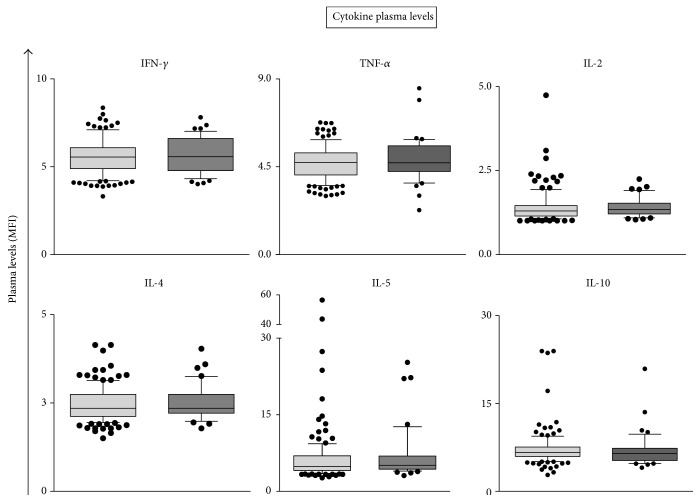
Cytokine plasma levels in group I (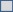) as compared to those in group II (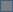). Plasma levels of proinflammatory (IL-2, TNF-*α*, and IFN-*γ*) and regulatory (IL-4, IL-5, and IL-10) cytokines were determined by cytometric bead array. Results are expressed in mean fluorescence intensity (MFI); data are presented in a box-plot format. The median is shown as a line across the box. Group I: HD patients without VAT. Group II: HD patients with episodes of VAT.

To characterize the overall cytokine pattern of hemodialysis patients with or without VAT, we have first compared plasma levels of cytokines according to the presence (group II) or absence of thrombotic events (group I). In these comparisons, no statistical difference was observed (Figure 1). We also did the comparison among groups regarding the ratio of pro- (IL-2, TNF-*α*, and IFN-*γ*) versus anti-inflammatory cytokine (IL-10), and no difference was found (data not shown).

**Figure 2:**
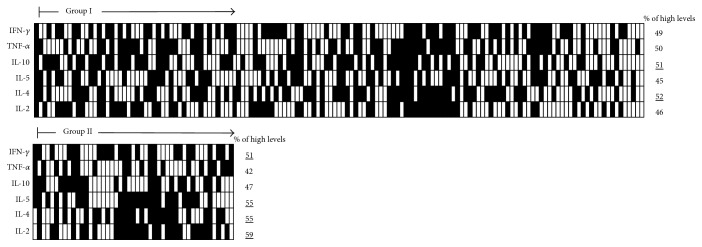
Multicytokine diagrams were used to quantify the frequency of HD patients with high levels of cytokines in the studied group. The global median value for each cytokine was used as the cut-off edge to tag each patient as they display “low levels” (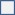 for all cytokines) and “high levels” of proinflammatory or regulatory (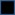 for IL-2, IL-4, IL-5, IL-10, TNF-*α*, and IFN-*γ*) cytokines. Group I: HD patients without VAT. Group II: HD patients with episodes of VAT.

In order to assemble the “cytokine signature” of each study group, the global median value was determined for each cytokine: IL-2 = 1.3, IL-4 = 2.4, IL-5 = 5.0, IL-10 = 6.6, TNF-*α* = 4.7, and IFN-*γ* = 5.6, all expressed in MFI. These values were used to establish the cut-off point used to separate patients with “low” or “high” cytokine levels. Using these criteria, each one received a tag for each cytokine. Following, diagrams were used to assemble the proinflammatory and anti-inflammatory profiles and to calculate the frequency (%) of patients showing “high cytokine levels” as showed in Figure 2.

**Figure 3:**
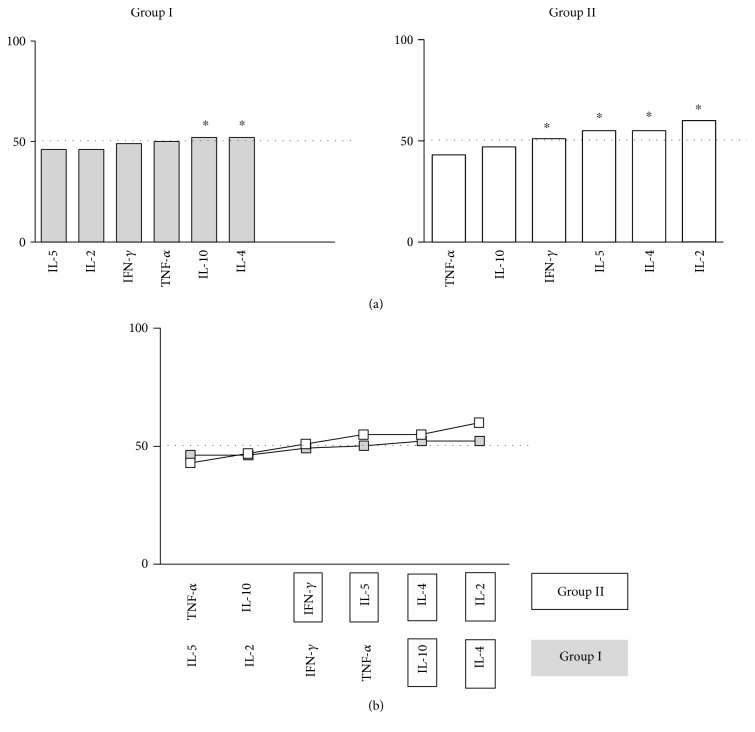
“Cytokine signatures” of HD patients without VAT and with VAT. (a) The ascendant frequency of HD patients with high levels of plasma cytokine was assembled and data expressed by bar graphs. (b) The cytokine signatures were further overlaid for HD patients to identify relevant elements in the cytokine signature that emerge above the 50th percentile (cut-off dotted line). Group I: HD patients without VAT. Group II: HD patients with episodes of VAT. ^∗^*p* < 0.05.

The frequency of patients with high cytokine levels was further compiled to establish the cytokine-ascendant profile, referred as “cytokine signature” for each study group (Figure 3(a)). Data analysis was carried out considering only the relevant cytokine frequencies above the 50th percentile. According to this approach, in patients who had a previous episode of VAT, the relevant components of the “cytokine signature” are the inflammatory cytokines, IL-2 and IFN-*γ*, and the modulatory Th-2 cytokines, IL-4 and IL-5. On the other hand, patients who have not experienced previous episode of VAT showed an increased frequency of only anti-inflammatory and modulatory cytokines, IL-10 and IL-4, respectively. The overlay of ascendant “cytokine signature” from the two study groups was further used to illustrate these findings (Figure 3(b)).

**Figure 4:**
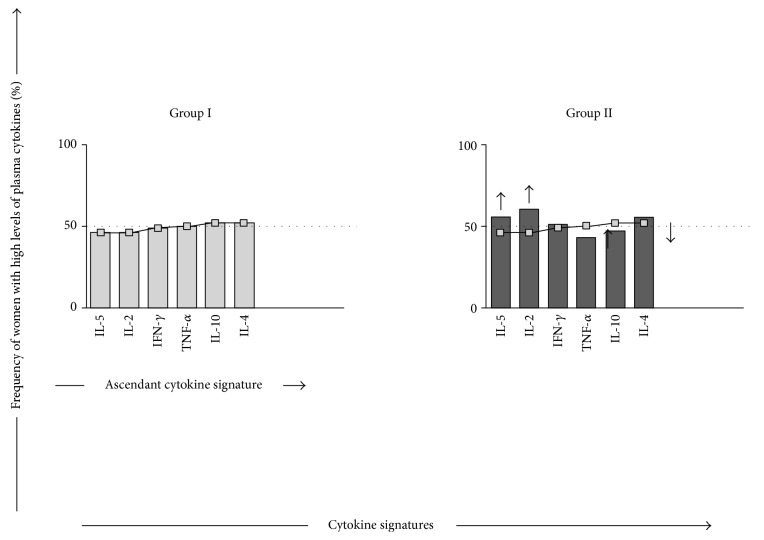
Comparative analysis of the cytokine signatures between group I (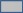) and group II (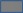). The ascendant frequency of group I with high cytokine plasma levels was assembled for group II as demonstrated by bar graphs and by the ascendant cytokine curve for group I (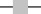). The ascendant frequency of group II with high cytokine plasma levels was also assembled for group I (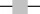) and used for comparative analysis with the cytokine profile of HD patients with VAT. Dotted lines indicate the 50th percentiles used as the cut-off to identify relevant elements, highlighted by ↑, for increased frequencies. Group I: HD patients without VAT. Group II: HD patients with VAT.

For better understanding of the results, the ascendant “cytokine signature” from patients who had no previous episode of VAT (group I) was used as a reference curve for comparative analysis with the patients who had a previous episode of VAT (group II) (Figure 4). Considering only the relevant cytokine frequencies above the 50th percentile, data analysis showed higher frequency of inflammatory cytokines, IL-2 and IFN-*γ*, and only one modulatory cytokine, IL-5, along with lower frequency of the anti-inflammatory cytokine, IL-10 (Figure 4).

## 4. Discussion

In the present study, the plasma levels of Th-1 and Th-2 cytokines were evaluated and a comparison was made between patients who previously presented VAT and those who did not. Indeed, no significant differences were detected in the comparison of both groups of ESRD patients on hemodialysis. It is important to highlight that, due to the cross-sectional design of our study, the evaluations were performed only in a single time point for all patients. Therefore, it is not possible to know if patients who did not exhibit previous VAT will have this complication in the future. Moreover, in our study, VAT was defined as a clinically evident episode of thrombotic occlusion. Thus, subclinical thrombosis was not considered. We believe that only slight differences would be expected in the comparison between the two groups of ESRD patients on hemodialysis. Indeed, all ESRD patients on hemodialysis have similar risk factors to develop VAT. It was not a surprise that we did not find differences in the comparisons of plasma levels of cytokines between patients who previously presented VAT and those who did not. For this reason, we performed the analysis of predominant cytokine profile of each group. The major advantage of applying the “cytokine signature” model for data analysis is the possibility to detect, with higher sensibility, putative minor changes in the cytokine profile not detectable by conventional statistical approaches [9].

The cytokine profile from each group showed that patients who did have VAT presented an increased frequency of inflammatory cytokines, IL-2 and IFN-*γ*, and an elevated frequency of modulatory IL-4 and IL-5, while for patients who did not present VAT, only the anti-inflammatory cytokine, IL-10, and the modulatory cytokine, IL-4, were increased (Figure 3). These data suggest that the presence of thrombotic events can be associated with higher proportion of inflammatory molecules, while, in the absence of these events, an anti-inflammatory and modulatory profile predominated.

In order to compare the impact of the presence of VAT in the profile of cytokines of the patients, the ascendant “cytokine signature” from the patients who have not experienced VAT was compared with patients that previously had VAT (Figure 4). In this way, only the cytokine frequencies above the 50th percentile were considered. The ascendant “cytokine signature” analysis revealed that the VAT group displayed an elevated percentage of patients with high levels of IL-2, IL-5, and IFN-*γ* and lower frequency of the anti-inflammatory cytokine, IL-10. These data suggest that thrombotic events may be associated with an unbalance between proinflammatory and anti-inflammatory cytokines.

Once thrombotic events in coronary arteries and deep limbs are associated with inflammation of the affected vessels, it can be considered that, in VAT, inflammation is involved in the thrombosis process [9–11]. However, the association between the presence of inflammation and complications of vascular access is still not very well established. In this regard, Liu et al. [18] observed that high-sensitivity C-reactive protein (hsCRP), TNF-*α* and IL-6 levels were significantly higher in hemodialysis patients who developed VAT (*n* = 14) when compared to the group of patients who did not have this complication (*n* = 18) and to the group of patients initiating hemodialysis (*n* = 15). The authors also observed that patients with VAT presented significant hyperplasia of the intimal layer of the vascular wall of the VAT and increased monocyte chemotactic protein (MCP-1) expression compared to those in the group that was initiating the treatment, and these alterations were positively correlated to increased levels of hsCRP. The authors hypothesized that microinflammation may be involved in the development of intimal hyperplasia, leading to stenosis and, consequently, VAT [19].

In response to vascular injury, smooth muscle cells undergo phenotypic changes characterized by increased affinity of integrins for their ligands, expression of adhesion molecules, cytokines, and growth factors, including IL-1, MCP-1, transforming growth factor beta 1 (TGF-*β*1), and TNF-*α* [18, 19]. These alterations facilitate leukocyte chemotaxis and vessel wall infiltration, as well as stimulate the expression of adhesion molecules on the endothelium and the production of extracellular matrix components, including collagen, elastin, and proteoglycans [20].

Chang et al. [21] observed abundant and moderate infiltration of macrophages and lymphocytes into the vascular appearance of VATs that developed thrombosis (*n* = 23) accompanied by increased expression of vascular adhesion molecule-1 (VCAM-1), proinflammatory cytokines (IL-6 and TNF-*α*), and matrix-9 metalloproteinase (MMP-9) compared to the vascular wall of vascular access that did not develop thrombosis (*n* = 13). The authors proposed that IL-6 and VCAM-1 might play a role in increasing proliferation of vascular smooth muscle cells and MMP-9, thus causing instability in the neointimal layer and subsequent injury of the endothelium, leading to thrombosis. They also suggested that anti-inflammatory treatment could be useful to improve the longevity of the vascular access.

Kourtzelis et al. [22] showed that complement activation triggered by hemodialysis biomaterials, and the subsequent generation of the complement anaphylatoxin C5a, resulted in the expression of functionally active TF in peripheral blood neutrophils [23]. Moreover, the persistent complement activation that occurs during long-term hemodialysis contributed to thrombosis in ESRD patients. The authors considered that complement contributed to the induction of granulocyte colony-stimulating factor, which has been implicated in the pathogenesis of thrombosis in patients treated with the recombinant form of this molecule, suggesting that the complement system could become a new therapeutic target for preventing thrombosis in patients on hemodialysis [23].

In summary, our data showed that the presence of thrombotic events in patients undergoing hemodialysis is associated with an elevated percentage of patients with high levels of proinflammatory cytokines (IL-2 and IFN-*γ*) and low levels of the anti-inflammatory cytokine (IL-10) that might drive the cytokine network in VAT patients towards an inflammatory state.

A study limitation of this study was that the time of vascular thrombosis occurrence and cytokines dosing was not to evaluat, and therefore, our findings only reveal associations and not causation due to the observational nature of the study. It should be pointed that longitudinal studies are needed to characterize the plasma cytokine profile in patients undergoing hemodialysis and its association with the occurrence of clinical complications, including thrombotic events. In addition, prospective studies might support the applicability of these biomarkers as prognostic tools for the follow-up of patients on hemodialysis.

## Figures and Tables

**Table 1:** Clinical characteristics and dialysis parameters of hemodialysis patients with (cases) and without (controls) previous episode of vascular access thrombosis.

Parameters	Group I (*n* = 149)	Group II (*n* = 46)	*p* value
Age (years)	52 (39–60)	50 (41–59)	0.380
Gender			0.193
Male [*n* (%)]	86 (58%)	20 (43%)	
Female [*n* (%)]	63 (42%)	26 (57%)	
BMI (kg/m^2^)	23.6 (21.1–26.5)	22.6 (20.1–28.5)	0.112
Cause of ESRD [*n* (%)]			
Hypertensive nephrosclerosis	51 (34%)	14 (30%)	0.633
Glomerulopathies	39 (26%)	7 (15%)	0.126
Diabetic nephropathy	21 (14%)	12 (26%)	0.060
Polycystic kidney disease	7 (5%)	3 (7%)	0.624
Others or unknowns causes	31 (21%)	10 (22%)	0.892
Predialysis arterial blood pressure			
Systolic blood pressure (mmHg)	130 (120–143)	140 (130–150)	0.060
Diastolic blood pressure (mmHg)	80 (80–90)	80 (80–90)	0.968
Type of vascular access			0.131
Arteriovenous fistula	144 (97%)	42 (91%)	
Arteriovenous graft	5 (3%)	4 (9%)	
Time on hemodialysis (months)	34.0 (17.0–90.3)	39.5 (19.0–92.0)	0.226
IWG (kg)	3.2 ± 1.1	2.9 ± 1.4	0.165
Medications [*n* (%)]			
Antihypertensive drugs			
ACE inhibitor	69 (46%)	69 (46%)	0.551
*β*-Blockers	64 (43%)	21 (46%)	0.747
Calcium channel antagonists	65 (44%)	17 (37%)	0.423
Acetylsalicylic acid	38 (26%)	8 (17%)	0.257
Statins	31 (21%)	5 (11%)	0.129
Insulin	29 (19%)	10 (22%)	0.736
Erythropoietin	129 (87%)	41 (89%)	0.651
Diabetes [*n* (%)]	40 (27%)	15 (33%)	0.448

The normally distributed data were expressed as mean ± SD (*t*-test). The non-Gaussian data were presented as median (range) (Mann–Whitney *U* test). Frequencies (%) were evaluated by *χ*2 test. Group I: patients without VAT. Group II: patients with VAT. BMI: body mass index; ESRD: end stage of renal disease; IWG: interdialytic weight gain.

**Table 2:** Laboratorial parameters of hemodialysis patients with (cases) and without (controls) previous episode of vascular access thrombosis.

Parameters	Group I (*n* = 149)	Group II (*n* = 46)	*p* value
Erythrocytes × 10^6^/mL	4.1 (3.6–4.5)	3.9 (3.6–4.2)	0.143
Hemoglobin (g/dL)	12.4 (11.1–13.8)	11.9 (10.7–13.1)	0.107
Hematocrit (%)	36.7 ± 5.7	35.7 ± 5.1	0.288
MCV	91.3 ± 5.6	91.3 ± 5.0	0.956
MCH	30.3 ± 2.2	30.0 ± 2.1	0.405
MCHC	33.3 (32.8–33.6)	33.2 (32.4–33.7)	0.440
Platelets × 10^3^/mL	210.0 (175.0–264.0)	224.5 (176.0–254.0)	0.693
Leukocytes × 10^3^/mL	7.0 (5.0–8.0)	6.2 (5.7–7.5)	0.685
Serum iron (*μ*g/dL)	58.0 (45.8–74.0)	54.0 (42.8–70.8)	0.443
TIBC (*μ*g/dL)	229.0 ± 48.0	226.0 ± 45.0	0.774
TS (%)	25.3 (19.0–34.0)	24.1 (20.0–31.0)	0.587
Ferritin (ng/mL)	340.0 (208.7–610.3)	319.5 (154.0–556.0)	0.198
TC (mg/dL)	164.3 ± 38.7	166.3 ± 34.5	0.747
LDLc (mg/dL)	93.2 ± 30.0	97.1 ± 25.0	0.440
HDLc (mg/dL)	34.0 (29.0–44.0)	33.0 (27.0–43.0)	0.304
TG (mg/dL)	150.0 (90.5–229.5)	126.0 (100.0–234.0)	0.983
hsCRP	3.7 (1.7–8.4)	3.2 (1.6–8.9)	0.811
Total protein (g/dL)	7.5 ± 0.7	7.3 ± 0.8	0.095
Creatinin (mg/dL)	12.0 ± 3.4	11.4 ± 3.0	0.322
Albumin (g/dL)	3.5 (3.3–3.8)	3.6 (3.4–3.8)	0.290
nPCR (g/kg/dia)	1.1 (1.0–1.4)	1.2 (1.0–1.6)	0.451

The normally distributed data were expressed as mean ± SD (*t*-test). The non-Gaussian data were presented as median (range) (Mann–Whitney test). MCV: mean corpuscular volume; MCH: mean corpuscular hemoglobin; MCHC: mean corpuscular hemoglobin concentration; TIBC: total iron binding capacity; TS: transferrin saturation; TC: total cholesterol; LDLc: low-density lipoprotein cholesterol; HDLc: high-density lipoprotein cholesterol; TG: triglycerides; hsCRP: high sensitivity C-protein reactive; nPCR: normalized protein catabolic rate.
